# Enhanced Photoluminescence and Prolonged Carrier Lifetime through Laser Radiation Hardening and Self-Healing in Aged MAPbBr_3_ Perovskites Encapsulated in NiO Nanotubes

**DOI:** 10.3390/mi14091706

**Published:** 2023-08-31

**Authors:** Steve Kamau, Roberto Gonzalez Rodriguez, Yan Jiang, Araceli Herrera Mondragon, Sinto Varghese, Noah Hurley, Anupama Kaul, Jingbiao Cui, Yuankun Lin

**Affiliations:** 1 Department of Physics, University of North Texas, Denton, TX 76203, USA; stevekamau@my.unt.edu (S.K.); roberto.gonzalezrodriguez@unt.edu (R.G.R.); yan.jiang@unt.edu (Y.J.); araceliherreramondragon@my.unt.edu (A.H.M.); sintovarghese@my.unt.edu (S.V.); noahhurley@my.unt.edu (N.H.); jingbiao.cui@unt.edu (J.C.); 2 Department of Materials Science and Engineering, University of North Texas, Denton, TX 76203, USA; anupama.kaul@unt.edu; 3 Department of Electrical Engineering, University of North Texas, Denton, TX 76203, USA

**Keywords:** nanofabrication, organic-inorganic perovskite, stability, enhanced photoluminescence, fluorescence lifetime imaging, self-healing, laser irradiation

## Abstract

Organic-inorganic perovskites hold great promise as optoelectronic semiconductors for pure color light emitting and photovoltaic devices. However, challenges persist regarding their photostability and chemical stability, which limit their extensive applications. This paper investigates the laser radiation hardening and self-healing-induced properties of aged MAPbBr_3_ perovskites encapsulated in NiO nanotubes (MAPbBr_3_@NiO) using photoluminescence (PL) and fluorescence lifetime imaging (FLIM). After deliberately subjecting the MAPbBr_3_@ NiO to atmospheric conditions for two years, the sample remains remarkably stable. It exhibits no changes in PL wavelength during UV laser irradiation and self-healing. Furthermore, exposure to UV light at 375 nm enhances the PL of the self-healed MAPbBr_3_@NiO. FLIM analysis sheds light on the mechanism behind photodegradation, self-healing, and PL enhancement. The results indicate the involvement of many carrier-trapping states with low lifetime events and an increase in peak lifetime after self-healing. The formation of trapping states at the perovskite/nanotube interface is discussed and tested. This study provides new insights into the dynamics of photo-carriers during photodegradation and self-healing in organic-inorganic perovskites.

## 1. Introduction

Halide perovskites, such as MAPbX_3_ and CsPbX_3_ (X = Cl, Br, and I), have attracted heavy interest due to their potential commercial application in pure color LED and photovoltaic devices, especially due to their solution-processing, low-temperature, and ink-printing-based low-cost production methods [1,2,3]. Their solar cell efficiencies have risen rapidly to 26.1% in a decade [4], comparable to traditional semiconductor-based photovoltaic devices in the market. For LED application, the quantum efficiency of bicomponent perovskite nanocomposites can reach nearly 100% [5]. The light extraction of LED can be improved through an incorporation of a bio-inspired nanostructure or moiré pattern in the device [6,7,8,9]. However, halide perovskites are not stable in environmental conditions. Their photostability and chemical stability are serious issues due to environmental stimuli, such as light, temperature, and moisture [4,10,11,12,13,14,15,16,17,18,19,20,21]. Halide perovskite solar cells without any protection and encapsulation degrade within 3−5 months [4]. In aspects of chemical stability, 2D organic-inorganic perovskites are more stable, however less efficient in device performance, than their 3D partners [22,23,24,25,26]. Surface passivation and encapsulation of halide perovskites in nanotubes or polymers have improved their stability [2,20,27,28,29,30,31,32,33,34,35]. Self-healing in halide perovskites has been reported as a capability to recover to their original conditions after degradation through overnight recovery or light soaking after UV irradiation damage [4,13,14,17,19,36,37,38]. Mechanisms of photo-instability and self-healing have been studied through the irradiation of UV, protons, electrons, and X-ray [4,12,14,15,17,36,39,40,41,42]. Light-accelerated ion migration, cationic dissociation, formation of meta-stable trap states or defects in shallow states, lattice expansion and contraction, or charge trapping have been proposed to explain the photo-instability [4,10,12,19,36,43,44]. Large-area lifetime imaging [27,38,45] has been used to characterize the microscopic defects in organic-inorganic perovskites; however, this technology has not been used to study excitons or carrier lifetimes related to photostability and self-healing.

Ideally, the thermal stability and photostability of halide perovskites can be studied in LED or photovoltaic devices [46]. However, extra electron (hole) transport layers and electrical contact layers may hinder a clear explanation of observed data [47]. In order to study the underlying mechanism for photostability, PL enhancement, and self-healing, we study MAPbBr_3_ encapsulated in NiO nanotubes (MAPbBr_3_@NiO) only. In this paper, we study the MAPbBr_3_@NiO through PL and FLIM measurements. The sample of MAPbBr_3_@NiO was purposefully left in the laboratory environment 2 years ago, exposed to light, moisture, and temperature fluctuations to test its stability. This aged sample has not degraded and is now exposed to UV laser continuously and left overnight for recovery. Not only does the self-healing of the sample return its PL to its original intensity observed but the PL is also enhanced after UV irradiation hardening. FLIM measurement reveals the formation of many trap states with lesser amounts but longer carrier lifetimes after UV exposure of the sample. The self-healed sample shows decreasing of trap states and recovery of lifetime events, however, with increased peak lifetime. This study leads toward a new vision of UV-light-induced carrier dynamics and PL enhancement.

## 2. Sample Preparation and Measurement Methods

MAPbBr_3_@NiO were prepared 24 months (about 2 years) ago and deliberately left exposed to atmospheric conditions without any cover to test their stability. The process of creating NiO nanotubes has been thoroughly explained in a previous reference [29]. In brief, porous ZnO/Ni(OH)_2_ core-shell nanowires were grown by immersing ZnO nanowire arrays in a nickel chloride solution for the adsorption of Ni^2+^ on the ZnO surface, followed by the hydrolysis to form Ni(OH)_2_ by immersing them in a sodium hydroxide solution. NiO was formed after annealing the Ni(OH)_2_ at a high temperature of 500 °C in air. Finally, the ZnO cores were dissolved in 1% HCl resulting in porous NiO nanotubes. The porous NiO nanotubes were used as a matrix for the infiltration of MAPbBr_3_ into their walls through nano-pores. Any excess MAPbBr_3_ precursors (1:1 mol ratio of MABr and PbBr_2_) on the nanotube walls were removed through spin coating at 6000 rpm, followed by baking the samples at 95 °C.

Structural characterization was performed using scanning electron microscopy (SEM, COXEM CX-200PLUS) and transmission electron microscopy (TEM, JEOL JEM-2100) at 200 kV.

The steady-state (PL spectrum was excited using a 375 nm laser (CrystaLaser; 6 mW; beam diameter (1/e^2^) of 1 mm), collected using a 20× objective lens (numerical aperture NA = 0.4) on a swivel mount of cage system (Thorlabs), and measured using a BaySpec spectrometer through an optical fiber at room temperature. The laser was incident onto the sample surface at an angle of 45 degrees.

FLIM and lifetime histogram measurements were conducted using a MicroTime 200 (PicoQuant) time-resolved confocal fluorescence microscope. A 20× objective lens (numerical aperture NA = 0.4) was used in the confocal microscope. Photon detection events were recorded and processed using a PicoQuant PicoHarp 300 time-correlated single-photon counting system. A Picoquant 405 nm (80 MHz) picosecond laser was used. A repetition rate of 40 MHz was used for all measurements, and an average power of 0.025 mW was employed. A 450 nm long-pass filter was used before the single-photon detector, and a band-pass filter (530–570 nm) was used to limit the potential PL from the silicon substrate [48]. Data acquisition was performed using SymPho Time 64, with a signal integration time of 2 ms for each pixel.

## 3. Results

**Figure 1:**
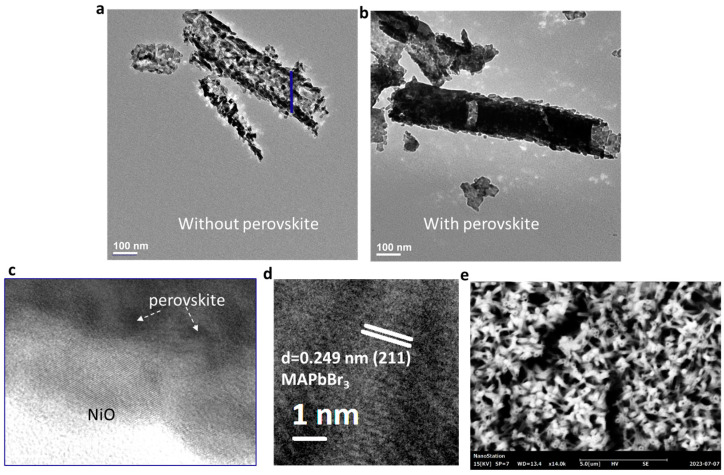
(**a**) TEM of NiO nanotubes without (**a**) and with (**b**) MAPbBr_3_. (**c**,**d**) High-resolution TEM of MAPbBr_3_@NiO (**c**) and a magnified TEM image of MAPbBr_3_ (**d**). (**e**) SEM of MAPbBr_3_@NiO.

Figure 1a,b show TEM images of NiO nanotubes without and with MAPbBr_3_ perovskites, respectively. The nanotube diameter is around 160 nm. The infiltrated nanotube looks less transparent. Figure 1c shows a high-resolution TEM image where the lattice corresponds to the (2,0,0) plane of NiO and dark spots are related to MAPbBr_3_ perovskites. A magnified TEM image is shown in Figure 1d where spacings (d = 0.249 nm) are visible in the lattice plane (2,1,1). Figure 1e shows an SEM image of 48-month-old NiO nanotubes filled with MAPbBr_3_ perovskites. The nanotubes are visible and have survived in the past two years. We conduct steady PL and time-resolved PL studies on these purposely aged samples below.

**Figure 2:**
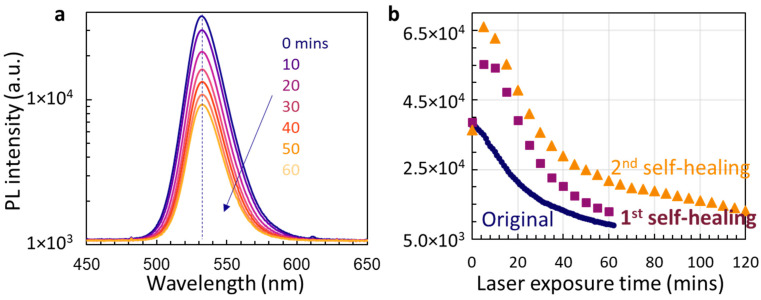
(**a**) Selective PL spectra of original MAPbBr_3_@NiO measured at 0, 10, 20, 30, 40, 50, and 60 min after the sample was exposed to the laser. (**b**) PL intensity as a function of laser exposure times for the original sample (blue symbols), the 1st PL measurement of self-healed samples after UV 375 nm laser is off overnight (purple squares), and the 2nd PL measurement of self-healed sample after second overnight (orange triangles).

First, we measure the PL every minute when UV laser 375 nm is continuously incident onto MAPbBr_3_@NiO. When the same setup was used for the PL measurement for 2D organic-inorganic perovskites without any protection, UV-laser-induced damage was observed after 20 min of UV exposure [32]. Figure 2a shows the PL spectrum of MAPbBr_3_@NiO after they receive continuous UV laser exposure for 0, 10, 20, 30, 40, 50, and 60 min. Although the PL intensity drops with increasing UV exposure times, the PL peak wavelength stays the same at around 532 nm as indicated by the dashed line in the figure. Organic-inorganic perovskites can have light-, temperature-, or moisture-induced degradation, especially degradation of MAPbI_3_ in vapor moisture [4,36,47]. Without contact with air and moisture for MAPbBr_3_@NiO, we only consider light-induced degradation. Prolonged exposure of organic-inorganic perovskites can result in the dissociation of the methylammonium ion [CH3NH3]^+^ [47]. From Figure 1a, we can conclude that there is no dissociation of the methylammonium ion [CH3NH3]^+^ or formation of PbBr_2_ because of the same PL peak wavelength at 532 nm in Figure 2a while PbBr_2_ has a PL wavelength at 521 nm [49,50].

We then check the PL intensity as a function of continuous UV exposure times. As shown in Figure 2b (blue circles), the first-day measurement shows a fast decrease in PL intensity from 0 to 30 min; however, the decreasing slope becomes smaller from 30 to 60 min. We then turn off the UV laser. After sitting the sample in the dark overnight, we measure it again. The first self-healing of MAPbBr_3_ perovskites enables the recovery of PL intensity to the original one (i.e., at 38,600, square symbol at 0 min). However, after 5 min of UV laser exposure, the PL intensity jumps from 38,600 to 55,200 (43% increase). Then, PL intensity drops from 55,200 to 12,900 after 60 min of continuous exposure to a 375 nm UV laser. Then, we turn off the UV laser. After a second overnight self-healing, the PL intensity (triangle symbol) recovers to the original one at 0 min and jumps by 81% after 5 min of UV laser exposure. From 5 to 120 min (about 2 h), PL intensity in the second self-healed measurement drops in a similar slope as the first self-healed case.

For each data set in Figure 2b (blue circles, purple squares, and orange triangles), the PL peak wavelength is the same. Overall, the PL peak wavelength is the same for all data points in Figure 2b.

**Figure 3:**
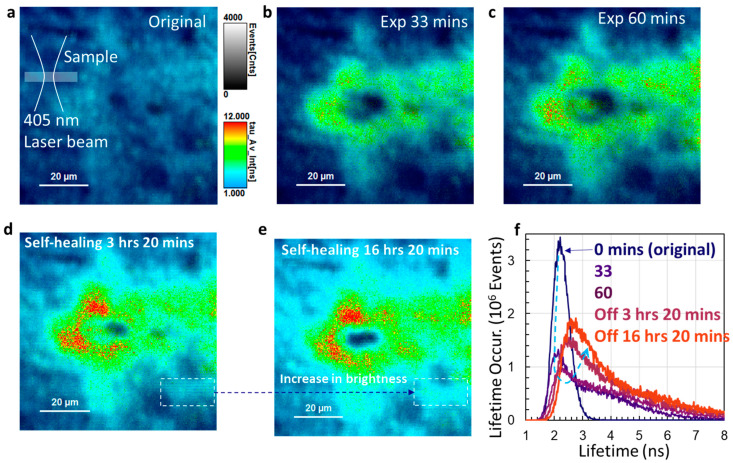
(**a**–**c**) FLIM of MAPbBr_3_@NiO after they are continuously exposed to 405 nm laser for 0 min (original sample), 33 min, and 60 min, respectively, at the focus of the laser beam. Inset in (**a**) shows the sample location in the laser beam. (**d**,**e**) FLIM of MAPbBr_3_@NiO after exposed samples are self-healed for 3 h-20 min and 16 h-20 min, respectively. (**f**) Lifetime histogram for all FLIMs in (**a**–**e**).

We then examine the reason PL intensity drops with prolonged UV laser exposure and PL intensity increases after self-healing, through FLIM measurement. A 405 nm laser was used, which is close to the 375 nm laser in wavelength. The inset of Figure 3a shows the exposer setup where the MAPbBr_3_@NiO are located at the focus of the objective lens. The same laser was used for the FLIM measurement. The same scale bar for events and lifetime (Tau, τ) is used for all FLIMs. Figure 3a–c show the FLIM of the MAPbBr_3_@NiO after they are exposed to a 405 nm laser for 0 min (original sample) and continuously exposed to the laser for 33 and 60 min. In Figure 3a, the lifetime is almost the same everywhere while the spatial lifetime events are determined by the filling fraction of NiO nanotubes. Following the lifetime scale bar, the carrier lifetime near the focus of 405 nm laser beams increases with the exposure times of the sample to the 405 nm laser.

Figure 3d,e show the FLIM of MAPbBr_3_@NiO after the laser was off for 3 h-20 min and the sample was left overnight in the dark (16 h-20 min after the laser was off). More carriers are trapped in states with higher lifetimes as judged by the lifetime scale bar. Lifetime events increase after self-healing overnight, as indicated by the dashed rectangles in Figure 3d,e. Figure 3f shows the lifetime histogram for all FLIMs in Figure 3. The carrier lifetime of the original sample has a Gaussian distribution with a central lifetime around 2.2 ns. After an exposure of 33 min, the carrier lifetime spreads from 1.6 ns to 7 ns. The carrier lifetimes in some areas are not modified at all as the short lifetime side of the curve still matches with the left side of the Gaussian curve from the original sample. After 60 min, the lifetime is shifted from the left side of the Gaussian curve to the right, and the lifetime-event peak drops further.

After the laser has been turned off and self-healed for 3 h-20 min and 16 h-20 min (overnight), the left side of the histogram curve shifts a little to a longer lifetime, the histogram peak increases (due to self-healing), and the peak lifetime shifts from 2.2 ns to 2.45 ns and further to 2.6 ns. However, there is no change in occurred events between the 4 ns and 8 ns lifetime ranges. These lifetime ranges are due to many carrier trap states. It indicates that sample degradation in certain areas cannot be recovered overnight after the sample is exposed to the focus of a 405 nm laser.

**Figure 4:**
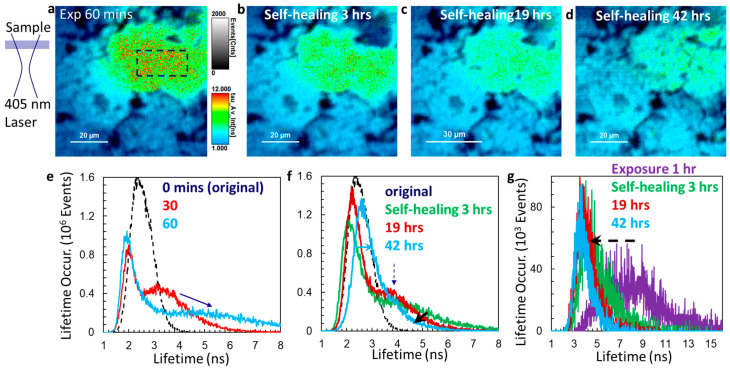
(**a**) Schematic of sample position within the laser beam and FLIM of MAPbBr_3_@NiO after they are continuously exposed to 405 nm laser for 60 min. (**b**–**d**) FLIMs of MAPbBr_3_@NiO after exposed samples are self-healed for 3 h, 19 h (overnight), and 42 h (second overnight), respectively. (**e**) Lifetime histogram of FLIMs after the samples are continuously exposed to 405 nm laser for 0 min (original sample; dashed blue line), 30 min (red line), and 60 min (light blue line). (**f**) Lifetime histogram of FLIMs after the exposed samples are self-healed for 3 h (green), 19 h (red), and 43 h (light blue). (**g**) Lifetime histogram of FLIMs for region of interest in the dashed rectangle (an example in (**a**)) for sample exposed for 1 h (purple) and self-healed sample for 3 h (green), 19 h (red), and 43 h (light blue).

The change in lifetime events for lifetimes above 4 ns is not observed between 3 h and 16 h self-healing processes in Figure 3. We then explore the self-healing process in the samples (but at a different location) when they are exposed to an expanded laser beam of 405 nm (the sample is defocused by one turn of the fine adjustment of the objective lens, about 0.2 mm). A schematic of the sample position within the laser beam is shown in Figure 4a. FLIMs of MAPbBr_3_@NiO are measured after the samples are continuously exposed to the 405 nm laser for 0 (original sample), 30, and 60 min and self-healed for 3 h, 19 h (overnight), and 42 h (second overnight). Figure 4a–d show the FLIM for the sample exposed for 60 min and self-healed samples for different hours. The lifetime histogram of FLIMs shows rich information related to degradation and self-healing. We separate them into three figures: Figure 4e for degradation, Figure 4f for the self-healing process, and Figure 4g for lifetime change at the region of interest which is the center region of laser exposure (dashed rectangle in (a); the same rectangle and location for other FLIMs). From Figure 4a–d, it is clearly observed that the red and yellow colors (defined as high lifetimes in the lifetime scale bar) become less dense from Figure 4a to Figure 4b and barely visible in Figure 4c,d.

From the lifetime histogram in Figure 4e, we can see that the lifetime events in the sample exposed for 30 min occur at the same level as the original sample between 1.5 and 2 ns. Above 2 ns, the lifetime events drop and form a broad secondary peak around 3.2 ns, and lifetime extends up to 7 ns. After 60 min exposure to the 405 nm laser, the lifetime events drop further as indicated by an arrow in Figure 4e, and lifetime extends above 8 ns, indicating many degradation-related carrier-trapping states. Self-healing is a reverse process for lifetime events and lifetime range. As indicated by a solid blue arrow in Figure 4f, the lifetime events drop between 4.5 and 8 ns after the self-healing and lifetime range drops below 6 ns after 42 h self-healing. However, the peak position of lifetime events shifts from 2.1 to 2.2 and to 2.6 ns during self-healing of 3, 19, and 43 h (about 2 days). Especially for the laser-exposed area in the region of interest isolated by the dashed rectangle in Figure 4, the histogram of lifetime shows a broad range of lifetime with a low event number for the sample exposed to UV for one hour in Figure 4g (purple line). During the self-healing, we see an increase in lifetime events and a shift in the central peak of lifetime events from 7 ns to 3.5 ns. The results in Figure 4 show a recovery of carriers from trapping states and a prolonged peak lifetime for carriers after self-healing. Comparing results in Figure 3 and Figure 4, we observe a shift in peak lifetime toward a higher number during the self-healing in both figures. We can observe a decrease in lifetime for these above 4 ns (recovering from trap states) during self-healing in Figure 4f,g; however, we do not observe it in Figure 3f.

## 4. Discussion

**Figure 5:**
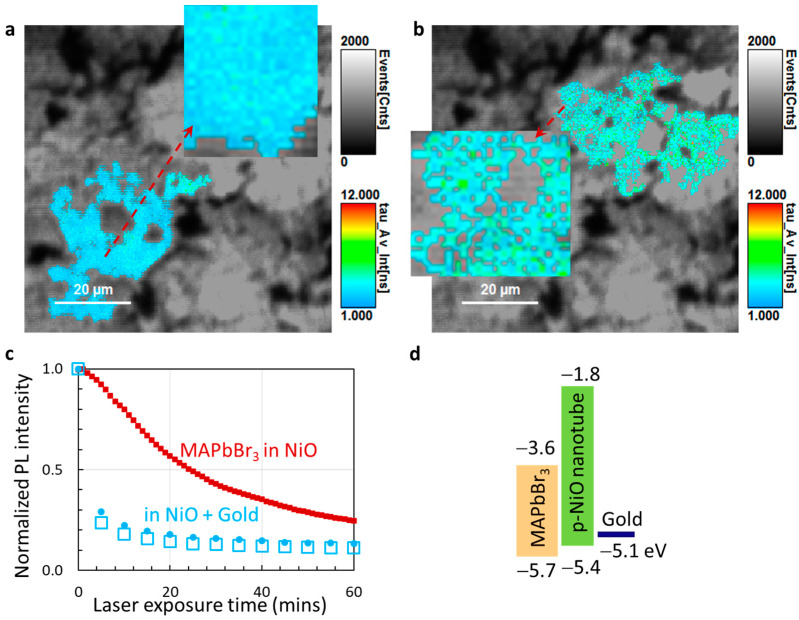
Regions of interest selected using “magic wand ROI” away from the laser exposure beam (**a**) and inside the exposure beam (**b**). Insets are enlarged views of areas indicated by dashed red lines. (**c**) Comparison of normalized PL intensity changes with UV laser exposure times for MAPbBr_3_@NiO (red squares) and gold-coated sample of MAPbBr_3_@NiO (blue circles and squares). (**d**) Band diagram for MAPbBr_3_ perovskites, p-type NiO, and gold film.

In the material system of MAPbBr_3_@NiO, there is a high percentage of perovskite contact with NiO, forming interfaces. Therefore, interface states (or edge states) play a role in photo-induced effects and can explain the carrier lifetime change. Using FLIM from Figure 4d, we check the spatial lifetime distribution using the magic wand region of interest (ROI) (a data process: mouse clicking activates all neighboring pixels with a similar fast lifetime in FLIM). Figure 5a shows the magic wand ROI near the corner of the FLIM (away from the 405 nm laser during one-hour exposure in Figure 4a). The inset in Figure 5a is an enlarged view of the ROI from a location indicated by the dashed red line. The FLIM in the inset shows a solid color covering in the lifetime events although there are spotted structures that might correspond to the nanotubes. The magic wand ROI inside the exposure laser beam is shown in Figure 5b. The inset in Figure 5b looks different from that in Figure 5a. There are many spotted colorings with holes, similar to the morphology of nanotubes facing straight up in SEM. It can be caused by the different lifetimes between carriers at interfaces and inside the bulk.

We can further test the interface effect by coating MAPbBr_3_@NiO with a 6.5 nm gold film. After the gold coating, PL intensity is measured with a 375 nm laser and increasing exposure times, as shown in Figure 5c in two measurements (blue circles and squares). Compared with the data from the sample without gold coating (red squares), the PL intensity of the coated sample drops quickly during the first 5 min and stabilizes after 20 min. It can be explained by the perfect alignment of the band diagram of MAPbBr_3_ perovskites, p-type NiO, and gold film in Figure 5d [29]. The light-generated holes can transport through p-type NiO and accumulate at the gold film. After reaching an equilibrium, the PL intensity is stabilized.

PL intensity from MAPbBr_3_@NiO as a function of laser exposure times from two pieces of a 24-month-old LED device is shown in Figure S1 for a comparison. The PL intensity initially drops and then increases with 375 nm laser exposure times. Although the results are not related to the study of the underlying mechanism of photostability and self-healing in MAPbBr_3_@NiO, they show that MAPbBr_3_@NiO is stable after 24 months in the device.

## 5. Conclusions

In summary, an aged MAPbBr_3_@NiO was re-examined and survived 2-year exposure to environmental stimuli. Furthermore, continuous UV laser exposure of the sample showed photodegradation and subsequent self-healing. The self-healed and laser-radiation-hardened sample demonstrated enhanced PL. Using FLIM, we spatially examined the carrier lifetime across the low- and high-exposure regions. The photodegradation was explained by the formation of numerous trapping states with low lifetime events, while the enhanced PL can be attributed to the prolonged peak lifetime observed in the lifetime histogram of the self-healed MAPbBr_3_@NiO.

## Data Availability

Data will be available upon request.

## Supplementary Materials

The following supporting information can be downloaded at: https://www.mdpi.com/article/10.3390/mi14091706/s1, Figure S1. Photoluminescence intensity from MAPbBr3 encapsulated in NiO nanotubes as a function of laser exposure times from two pieces of LED device. The structure of LED is described in the reference [29]. The LED device was fabricated 24 months ago, run for 140 h, and was cut into pieces for SEM examination of the cross section of LED device for the reference paper [29].
