# Supplementary material for: Enhanced Photoluminescence and Prolonged Carrier Lifetime through Laser Radiation Hardening and Self-Healing in Aged MAPbBr3 Perovskites Encapsulated in NiO Nanotubes
Source: Micromachines (Basel). 2023 Aug 31;14(9):1706. doi: 10.3390/mi14091706 (PMC10534348; doi:10.3390/mi14091706)
Supplement: Supplementary file 1 [file micromachines-14-01706-s001.zip › micromachines-2559589-supplementary.pdf]

Supporting information

Enhanced Photoluminescence and Prolonged Carrier Lifetime through Laser Radiation Hardening and Self-Healing in Aged $MAPbBr_3$ Perovskites Encapsulated in NiO Nanotubes

Steve Kamau [1], Roberto Gonzalez Rodriguez [1], Yan Jiang [1], Araceli Herrera Mondragon [1], Sinto Varghese [1], Noah Hurley [1], Anupama Kaul [2,3], Jingbiao Cui [1] and Yuankun Lin [1,3,*]

[1] Department of Physics, University of North Texas, Denton, TX 76203, USA; stevekamau@my.unt.edu (S.K.); roberto.gonzalezrodriguez@unt.edu (R.G.R.); yan.jiang@unt.edu (Y.J.); araceliherreramondragon@my.unt.edu (A.H.M.); sintovarghese@my.unt.edu (S.V.); noahhurley@my.unt.edu (N.H.); jingbiao.cui@unt.edu (J.C.)
[2] Department of Materials Science and Engineering, University of North Texas, Denton, TX 76203, USA; anupama.kaul@unt.edu
[3] Department of Electrical Engineering, University of North Texas, Denton, TX 76203, USA
* Correspondence: yuankun.lin@unt.edu; Tel.: +1-940-565-4548

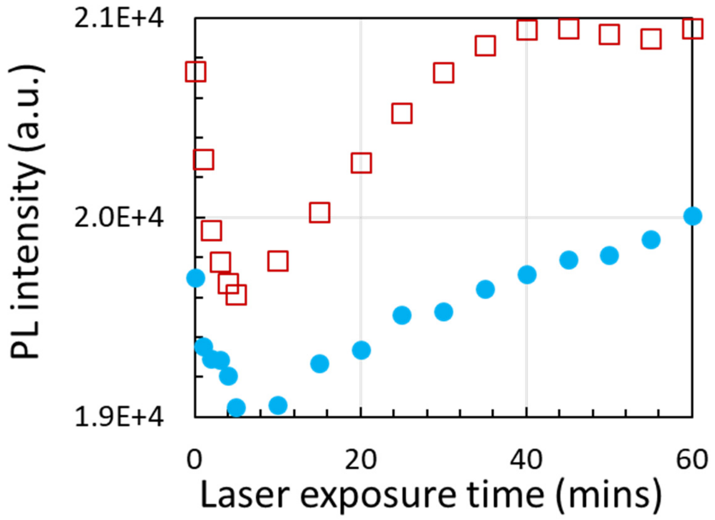

Figure S1. Photoluminescence intensity from $MAPbBr_3$ encapsulated in NiO nanotubes as a function of laser exposure times from two pieces of LED device. The structure of LED is described in the reference [1]. The LED device was fabricated 24 months ago, run for 140 hours, and was cut into pieces for SEM examination of the cross section of LED device for the reference paper [1].

1. Gonzalez-Rodriguez, R.; Hathaway, E.; Lin, Y.; Coffer, J.L.; Cui, J. Encapsulated $MAPbBr_3$ in Nickel Oxide Nanotubes and Their Electroluminescence. *Nanoscale* **2022**, *14*, 6417–6424, https://doi.org/10.1039/D2NR00019A.
